# Short-Term Safety and Tolerability of an Antimalarial Herbal Medicine,* CoBaT-Y017* in Healthy Volunteers

**DOI:** 10.1155/2019/7610476

**Published:** 2019-04-21

**Authors:** Adrien N. Noudjiegbe, Adeline L. Gnimassou, Judith S. Gbenoudon, Jean-Eudes Degbelo, Aurel C. E. Allabi

**Affiliations:** ^1^Faculty of Health Sciences, Laboratory of Pharmacology and Toxicology, University of Abomey-Calavi, Benin; ^2^Beninese Center of Scientific Research and Innovation, National Laboratory of Narcotic and Toxicology, Benin; ^3^Faculty of Sciences and Technic, Laboratory of Immunology of Infectious diseases and Allergic, University of Abomey-Calavi, Benin; ^4^Teaching Hospital of Abomey-Calavi/Sô-Ava, Laboratory of Biomedical Analysis, Benin; ^5^Service of medicine, Teaching Hospital of Abomey-Calavi/Sô-Ava, Benin

## Abstract

**Background:**

Malaria is the most prevalent parasitic disease in Benin and the main cause of morbidity and mortality. To fight this disease, a large proportion of the population resorts to herbal drugs. However, for most of these herbal preparations, no scientific evidence of their safety or efficacy has yet been established. The aim of this study was to evaluate the short-term safety and tolerability of * CoBaT-Y017* and collect some data on its antimalarial efficacy.

**Material and Methods:**

* CoBaT-Y017 *was formulated into syrup accommodated in 70 mL bottles. The trial involved a sample of 10 male volunteers, selected using the Lot Quality Assurance Sampling (LQAS) method and declared apparently healthy by a physician through clinical examination. During the baseline analysis, two cases of parasitaemia were detected. The volunteers were hospitalized for 5 days and orally given 35 mL of * CoBaT-Y017* diluted in 1.5 L of mineral water, for four consecutive days. Safety and tolerability were monitored clinically, haematologically, biochemically, and parasitologically on days 0 to 5, 7, and 14. Adverse events were recorded by self-reporting or by a physician through clinical examinations and biological investigations.

**Results:**

60% of the volunteers experienced no adverse events; appetite increase (40%) and drowsiness (20%) were adverse events noted. There were no changes in physical characteristics or vital signs and haematological and biochemical parameters. The two initial positive cases of parasitaemia became negative 24 hours after administration.

**Conclusion:**

* CoBaT-Y017* presented a significant safety and tolerability in healthy volunteers to allow its further development by starting a phase II clinical study.

## 1. Introduction

The resurgence of malaria due to drug-resistant strains of the parasite and insecticide-resistant strains of the mosquito vector retains the attention of many scholars. As a result, there have been various efforts to combat the problem of parasite resistance, such as reversing chloroquine resistance, using combination therapy, and discovering new antimalarial compounds from various sources, especially from traditional medicinal plants [1, 2].

Medicinal plants have contributed significantly to current malaria treatment. Artemisinin and quinine, main antimalarial drugs today, are provided by medicinal plants, but artemisinin-resistant parasite strains are emerging [3]. The fight against malaria still requires the discovery of effective, well-tolerated medicines [4].

There is much research on traditional herbal medicines in Africa, but very little of it is clinical. In a review, out of more than 1200 plant species reportedly used for the treatment of malaria, only 13 have undergone clinical trials, although hundreds have been tested in the laboratory [5].

Furthermore, in Africa, there are many herbal remedies available on the market but with no clinical trials establishing their safety and efficacy. This situation is due to the insufficient implementation of regulation on medicines and especially on phytomedicines, which is quite common in many developing countries. In order to fill this gap, leading to standardized herbal medicine which will be officially authorized on the market, a “reverse pharmacology” approach is being used, where clinical evaluation is prioritized. The primary objective of this approach is to improve the utilization of herbal medicines, which are already in use and in the process of being approved [4, 6].

In Benin, many antimalarial herbal medicines have been developed and are widely used by the population. Clinical information was collected retrospectively on those remedies. In a survey conducted by Dr. Allabi,* CoBaT-Y017* was ranked as the leading phytomedicine antimalarial used in Benin for uncomplicated malaria self-treatment. According to consumers,* CoBaT-Y017* has less adverse events compared to other phytomedicines and would be the most effectiveness (personal communication, Dr. Allabi). Furthermore* CoBaT-Y017* has been commercially available and used in Benin and other countries since 2007 without marketing authorization. It has not been subject of any toxic or adverse events reported to the attention of the health authorities of those countries. Therefore, for its preclinical studies, a limit test was chosen in accordance with paragraph 19 of Organization for Economic Cooperation and Development (OECD) Guideline 423 [7]. After 14 days of acute toxicity study follow-up, the* CoBaT-Y017* single dose of 2000 mg/kg body weight did not cause any toxic symptoms or mortality. No significant changes were noted in the rats' behavior, water and food consumption, respiratory rate, and hair appearance, compared to control rats. In addition, no gastrointestinal manifestation of salivation, diarrhea, or constipation has been observed [8]. Also after 90 days of subacute toxicity study follow-up according to the OECD 408 protocol [9], no change in behavior, no toxic manifestations, and no deaths were registered in the rats under study. It was noted a complete organs architecture preservation in all test rats [8].

*CoBaT-Y017*'s good preclinical data obtained during the acute and subacute toxicity tests was submitted for phase I clinical trial.

The purpose of phase I clinical trial was to establish* CoBaT-Y017* short-term safety and tolerability in healthy adult volunteers, by the evaluation of clinical and biological parameters and detecting the occurrence of any emergent adverse events due to its administration.

## 2. Material and Methods

### 2.1. Description of the Herbal Drug

**Figure 1:**
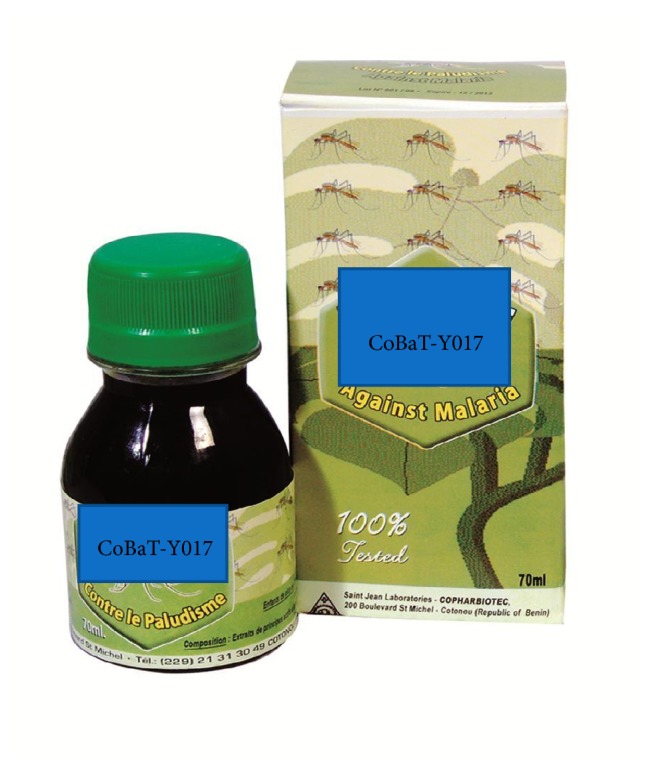
Commercial presentation of * CoBaT-Y017*.

*CoBaT-Y017* was prepared in Benin by COPHARBIOTECH, local manufacturer. The study was conducted using its commercial form, coffee-brown syrup accommodated in 70 mL bottles in a package including a measuring container of 10 mL (Figure 1).

### 2.2. Study Design and Sampling

Phase I clinical trial was a simple, one-arm prospective evaluation of safety and tolerability in ten (10) apparently healthy male volunteers aged 18 to 40, with negative or positive parasitaemia. They were selected using the Lot Quality Assurance Sampling (LQAS) method and were enrolled if they complied with a number of inclusion and exclusion criteria. The recruitment of volunteers has been among the population of Abomey-Calavi; all have given a written consent after being informed. The information sheet and the consent form were both in conformity with the International Guidelines for Research Involving Human Subjects of the WHO, and the Declaration of Helsinki on medical research involving human subjects. After obtaining the signed consent forms, the volunteers were taken for further examinations to determine the baseline of their biological parameters. Safety and tolerability were clinically monitored by comparison of vital signs, physical characteristics, and haematological and biochemical parameters at days 0, 1, 2, 3, 4, 5, 7, and 14, to the respective reported data. Biochemical parameters were determined using the CHEM-7 Cerba® Mannheim spectrometer and the automatic haematology analyzer HORIBA® ABX Micros ES60 was used for haematological parameters.

The trial was conducted from February 2017 to April 2017 at the Teaching Hospital Center of Abomey-Calavi/Sô-Ava in the Department of Internal Medicine and the evaluation of haematological and biochemical parameters as well as parasitaemia was carried out in the biomedical analysis laboratory of this hospital.

### 2.3. Inclusion Criteria

These are being male, aged between 18 and 40, being apparently healthy (this means that those subjects were not feverish and had no other signs of malaria with positive or negative parasitaemia) as observed by physical examination and haematological and biochemical investigations, no chronic pathology, no gastrointestinal intolerance including nausea, vomiting, and/or diarrhea, absence of any complaints before the treatment, no allergy to the constituents of * CoBaT-Y017*, no medical or herbal treatment of any kind in the past month, no substance abuse in the past month, no alcohol intake in the past week, being fit, and willing to come back for monitoring.

### 2.4. Exclusion Criteria

These include development of severe malaria according to WHO definition during the study, having an evidence of significant clinical abnormalities detected by a physician after inclusion, any kind of medical or herbal treatment intake during the study without the investigator consent, and alcohol intake during the study.

### 2.5. Drug Administration and Monitoring

35 mL of* CoBaT-Y017 *diluted in 1.5 L of mineral water was administered orally each day for 4 consecutive days. For this study the dilution of 35 ml of CoBaT-Y017 in 1.5L of water per day for four consecutive days is an approach that has been used for years in the treatment of uncomplicated malaria and that we did not want to deviate from.

This dose corresponded to 42.86 mg/kg body weight, which is significantly lower than the nontoxic 2000 mg/kg dose used during the preclinical studies [8]. The participants were hospitalized during the treatment and also on the fifth day to ensure strict monitoring. After five days in hospital, volunteers were reviewed at day 7 and day 14. At each visit (before new administration of the product) constants and complaints were recorded and a clinical examination was performed. Blood and urine samples were also collected to assess biological parameters.

### 2.6. Safety and Tolerability Monitoring

Clinically, the safety of the product was assessed through the vital signs such as blood pressure, pulse and respiratory rate. The adverse events were documented daily using a checklist [10]. They were recorded after either self-reporting by the participant or detection of clinical signs and changes in biological parameters by a physician during clinical examination. The adverse events were classified in accordance with the Common Terminology Criteria for Adverse Events (CTCAE) [11].

Tolerability was investigated through the monitoring of biological parameters using blood and urine samples at days 1, 2, 3, 4, 5, and 7. The main criteria to evaluate biological tolerability of CoBaT-Y017 were based on blood chemical which includes hepatic function test such as ASAT (12 to 42 IU/L) and ALAT (10 to 48 IU/L), renal function tests such as creatinine considered normal, when the value was between 7 and 13 mg/L, and haemoglobinuria and proteinuria whose absence in the urine were considered as normal. The glycaemia (Gly) was also monitored in which normal levels ranged from 3.3 to 6.1mmol/L as well as the prothrombin (TP) rate in which normal value is at least 72%. For complete blood numeration, the haematological parameters were considered normal when haemoglobin rate (Hb) is 13 to 17 g/dl, red cell number (NR) is 4.2 to 5.7 (X10^6^ per mm^3^), hematocrit rate is 40 to 50%, mean corpuscular volume is 85 to 95 fl, mean corpuscular concentration in haemoglobin is 32 to 36%, platelet number (Plat) is 150 to 400 (X10^3^ per mm^3^), leukocyte number (NW) is 4 to 10 (X10^3^ per mm^3^), neutrophil rate is 40 to 75%, lymphocyte rate is 20 to 45%, eosinophil rate is 1 to 4%, basophil rate is 0 to 1%, and monocyte rate is 2 to 8%.

All parameters were compared to the baseline values [12]. The evaluation of therapeutic efficacy was based on parasitaemia clearance, through the thin smears made at each visit until day 14.

### 2.7. Statistical Considerations and Data Analysis

Data treatment forms were created to collect clinical and biological data for each volunteer. The data collection forms included the clinical follow-up and the biological follow-up. At the end of the data collection, all data of each participant was brought together in case record forms.

The data was entered in Microsoft Office Excel 2013 and transferred to Stata 11 and SPSS 16.0 for further analysis. The data was expressed as mean ± standard deviation and/or standard error when needed. Comparison of different parameter means over time was carried out using Friedman*ʼ*s test. A p value ≤ 0.05 was considered as significant.

### 2.8. Ethical Considerations

This study protocol was approved by the Research Ethics Committee of Applied Biomedical Sciences Institute (CER-ISBA) of Cotonou at this reference: N°103 du 09/01/17. The clinical trial was conducted in compliance with the Declaration of Helsinki on medical research involving human subjects, the Good Clinical Practice (GCP) guidelines, and the Standard Operating Procedures elaborated by the UNDP/World Bank/WHO/TDR [10, 13]. All samples were coded with an initial and a specific number for each participant. All participants had given a signed written consent after being informed and had the freedom to withdraw from the study at any time.

## 3. Results 

### 3.1. Baseline of Participants' Physical and Clinical Characteristics

Ten volunteers were recruited to participate in this phase I clinical trial study, based on the inclusion and noninclusion criteria.

The median age of participants was 25 years with extremes of 22 and 35 years and only men in this age group were enrolled. The average body mass index (BMI) of these volunteers was 19.91 ± 2.01 kg / m^2^. This was a normal BMI value and corresponded to subjects of normal weight. Only one participant had a BMI below 18 kg/m^2^ and was considered as a lean subject. However, the physical examination of this volunteer, as for the others, did not show any abnormalities or pathological features.

At inclusion, no abnormalities were observed for the pulse of volunteers whose mean at baseline was 66.00 ± 3.06 beats per minute. The mean systolic blood pressure (SBP) of subjects at baseline was 12.00 ± 0.67 cmHg and the mean diastolic blood pressure (DBP) was 7.00 ± 0.94 cmHg. As these values are within the normal limits, all volunteers therefore had normal blood pressure at inclusion. These volunteers also had normal temperatures; mean at baseline was 36.4 ± 0.7°C.

### 3.2. Baseline of Participants' Biological Parameters 

Overall, the participants' haematological and biochemical parameters were normal at inclusion. The haemoglobinuria and proteinuria tests were also negative for all volunteers at inclusion, thus showing that none of the volunteers suffered renal or hepatic disease.

### 3.3. Product Appreciation

20% of volunteers found * CoBaT-Y017 *to have a pleasant taste, 60% considered it to have a good taste, and 20% thought it has an acceptable taste.

### 3.4. Evolution of the Volunteers' Pulses

**Figure 2:**
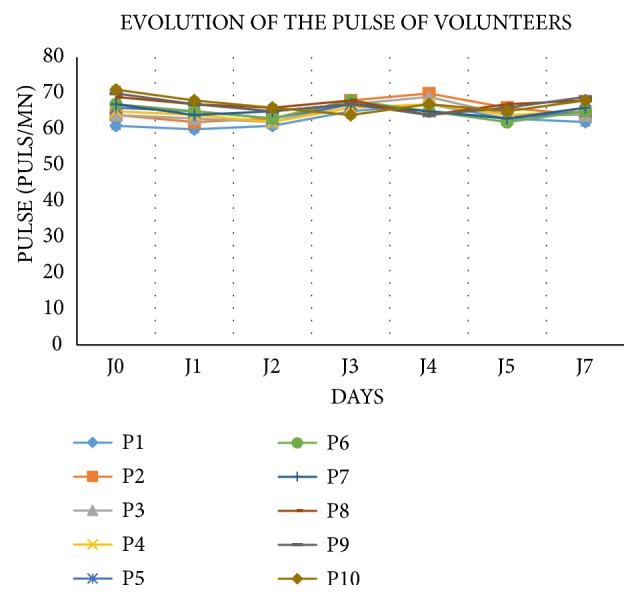
Pulse evolution during the monitoring period.* Legend: P: participant; J: day*.

The pulse values of the study subjects remained within normal limits during the monitoring period. Nonsignificant changes were observed between consecutive days after administration and until day 14 (Figure 2).

### 3.5. Evolution of Systolic and Diastolic Blood Pressures

Systolic and diastolic blood pressures remained normal throughout the monitoring period and did not show significant variation.

**Figure 3:**
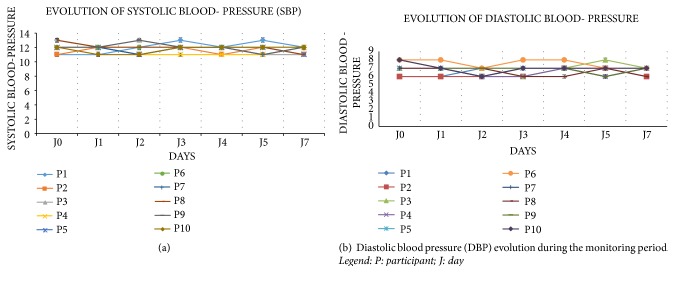


Figure 3(a) shows nonsignificant change observed in the participants' systolic blood pressure which remained within the normal limits after the start of the treatment and throughout the monitoring period.

Figure 3(b) shows nonsignificant difference between days for the participants' diastolic blood pressure after the start of the treatment; it remained between 6 and 8 cmHg.

### 3.6. Evolution of Temperature

**Figure 4:**
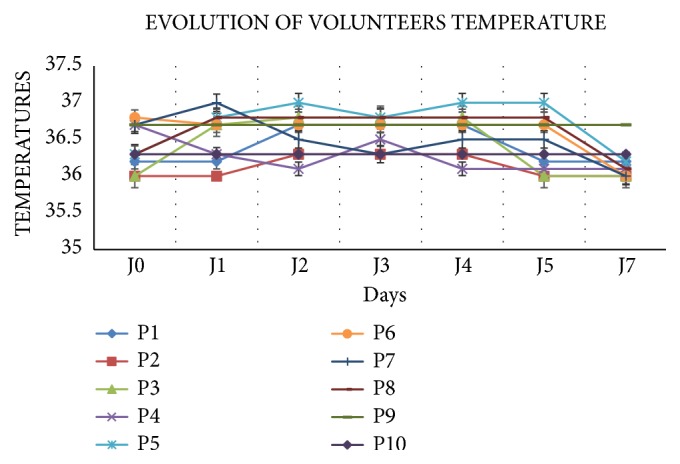
Evolution of volunteers' temperature during the monitoring period.* Legend: P: participant; J: day.*

We observed nonsignificant variation of temperature throughout the monitoring period. The participants' temperature remained between 36°C and 37°C until day 7 after the start of the treatment (Figure 4).

### 3.7. Haematological Tolerability

Table 1 shows the mean values and ranges of haematological parameters at inclusion until day 7. We observed after the start of treatment, and throughout the monitoring period, nonsignificant variation between days in the progression of all haematological parameters of the participants (P>0.05). Nonsignificant differences between days (P=0.23) were observed in the prothrombin progression after the start of treatment.

### 3.8. Hepatic and Renal Tolerability

Table 2 shows the mean values and ranges of creatinine, glycaemia, and transaminases GOT and GPT. The mean values of creatinine and glycaemia remained almost constant throughout the monitoring period while nonsignificant increase (P>0.05) was observed between days for the mean values of transaminases GOT and GPT, after the start of treatment.

Daily haemoglobinuria and proteinuria tests remained negative during the monitoring period.

According to these results, * CoBaT-Y017 *disturbed neither the hepatic function nor the renal function of the participants.

### 3.9. Activity of * CoBaT-Y017* on Parasite Clearance

Table 3 shows the product activity observed during this study. Two volunteers out of the ten (10) presented positive parasitaemia, 8700 and 380 parasites per microliter, respectively, at day 0. Their parasitaemia turned negative 24 hours after * CoBaT-Y017* administration and remained negative during the entire monitoring period, until day 14.

### 3.10. Adverse Events

During the treatment, 60% (six out ten) of volunteers said they felt no adverse events, while 20% (two out of ten) reported both increased appetite and drowsiness, and another 20% reported increased appetite only. However, all adverse events disappeared at once with the end of the treatment (day 5).

According to the classification of adverse events of CTCAE, the two events recorded (increased appetite and drowsiness) are categorized as grade 1 adverse events.

## 4. Discussion

Our phase I study fills part of the scientific gap [5] and contributes to the medical evidence of a herbal drug. Indeed, we reviewed several clinical and biological parameters following the administration of our investigated product, * CoBaT-Y017,* in apparently healthy adult male volunteers. Preclinical data on reproduction toxicity are not available. For precautionary reasons, women were not included, although surveys have found women using this product.

Results of this study revealed no variation of hepatic transaminases profile over the normal limits after administration of the product. This result is similar to what was observed with another antimalarial herbal medicine, the extract of* Nauclea pobiguinii *[14]. Also, this hepatic profile of * CoBaT-Y017* was proven safer than that of the extract of* ELAEIS GUINEENS *JACQ [15], another Beninese antimalarial herbal medicine.

The absence of serious adverse events corroborated the results obtained in a study on a plant whose extract is one of the compounds of our investigated product [16]. Furthermore the global safety profile exhibited by * CoBaT-Y017* is much better compared to the extract of* ELAEIS GUINEENS *JACQ widely dispensed in Benin [15]. For this latter product, almost all the volunteers treated exhibited adverse events compared to 40% for * CoBaT-Y017*. Diarrhea and asthenia were the two major side-effects observed for the extract of* ELAEIS GUINEENS *JACQ, whereas some extracts contained in * CoBaT-Y017* were known for their antiasthenic activity [16]; this would avoid such side-effects and would probably help shorten the convalescence period. Asthenia was also reported with a significant frequency (7%) for the extract of another antimalarial medicinal plant,* Nauclea pobiguinii* [14], compared to 0% in our study. No cases of headaches were reported for * CoBaT-Y017* compared to 23% for* Nauclea pobiguinii* [14]. Appetite increase was reported, at 40%, in our study compared to 33% for* Nauclea pobiguinii* and drowsiness was observed, also at 20%, for * CoBaT-Y017* compared to 7% for* Nauclea pobiguinii* [14]. Moreover, the increased appetite would be favorable to fight anorexia, one of the symptoms of uncomplicated malaria [17]. There were neither life-threatening adverse events reported in this trial nor clinically significant adverse findings on vital signs, significant modification of biochemical, or haematological parameters attributable to * CoBaT-Y017*. This tolerability profile of * CoBaT-Y017* was in line with its preclinical studies which proved no toxicity [8].

The main interest of this work was that we started from the realities of field, remedy used informally and without marketing authorization, to appreciate the safety and the tolerability of use of the * CoBaT-Y017*. The evaluation of its effectiveness should be the next step. It is therefore a simplified approach called reverse pharmacology to develop at lower cost and faster an antimalarial phytomedicine [5]. 

## 5. Conclusion 

Our results demonstrate no significant adverse events and toxicity of * CoBaT-Y017*, including hepatotoxicity and renal toxicity. The product demonstrated good safety and tolerability in healthy volunteers to allow its further development by starting a phase II clinical trial.

## Figures and Tables

**Table 1:** Effects of* CoBaT-Y017* administration on evolution of haematological parameters.

Haematological parameters	Inclusion D0	Product Administration Days				Observation Days		Pvalue
		D1	D2	D3	D4	D5	D7	
Hb	14.2 ± 0.6	14.2 ± 0.6	14.0 ± 0.7	14.1 ± 0.9	14.1 ± 0.8	14.1 ± 0.7	14.0 ± 0.8	0.46
NRx10^9^	4.9 ± 1.8	5.1 ± 0.6	5.1 ± 0.5	5.2 ± 0.7	5.1 ± 0.6	5.0 ± 0.6	5.0 ± 0.7	0.50
NWx10^3^	4.9 ± 0.9	4.9 ± 0.6	5.0 ± 1.3	4.9 ± 0.6	5.0 ± 0.7	4.9 ± 1.3	4.8 ± 0.7	0.87
Plat.x10^5^	2.0 ± 0.4	2.4 ± 0.6	2.2 ± 0.6	2.1 ± 0.6	2.2 ± 0.6	2.2 ± 0.6	2.1 ± 0.5	0.18
TP	74.8 ± 10.8	72.4 ± 10.7	73.4 ± 11.0	73.4 ± 9.5	70.0 ± 12.0	77.1 ± 12.0	73.7 ± 13.7	0.23

Legend: D: day; Hb: haemoglobin; NR= number of Red cells; NW= number of white cells; Plat: platelet; TP: prothrombin rate.

**Table 2:** Effects of *CoBaT-Y017* administration on the evolution of biochemical parameters.

biochemical parameters	Inclusion D0	Product Administration Days				Observation Days		p value
		D1	D2	D3	D4	D 5	D 7	
CREA	10.4 ± 1.6	10.4 ± 1.6	10.0 ± 2.1	10.1 ± 3.3	10.5 ± 2.1	10.5 ± 2.2	10.1 ± 1.8	0.46
TGO	24.5 ± 6.1	26.1 ± 7.8	26.5 ± 5.2	28.5 ± 7.3	27.6 ± 11.4	30.0 ± 22.0	29.2 ± 18.5	0.56
TGP	17.8 ± 5.3	18.5 ± 5.6	21.0 ± 11.4	22.0 ± 6.2	21.1 ± 6.3	22.5 ± 7.8	19.6 ± 11.2	0.87
GLY	0.8 ± 0.1	0.8 ± 0.1	0.8 ± 0.1	0.8 ± 0.1	0.7 ± 0.1	0.7 ± 0.1	0.8 ± 0.1	0.28

Legend: D: day; CREA: creatinine; TGO: transaminase GO; TGP: transaminase GP; GLY: glycaemia.

**Table 3:** Activity of *CoBaT-Y017* on parasite clearance.

patients	Inclusion D0	*Paludose®* Administration Days				Observation Days		
		D1	D2	D3	D4	D5	D7	D14
P1	-	-	-	-	-	-	-	-
P2	-	-	-	-	-	-	-	-
P3	-	-	-	-	-	-	-	-
P4	+8700 p/*μ*L	-	-	-	-	-	-	-
P5	-	-	-	-	-	-	-	-
P6	-	-	-	-	-	-	-	-
P7	-	-	-	-	-	-	-	-
P8	-	-	-	-	-	-	-	-
P9	+380p/*μ*L	-	-	-	-	-	-	-
P10	-	-	-	-	-	-	-	-

Legend: P: participant; D: day; +: presence of trophozoites of *Plasmodium falciparum;* -: absence of trophozoites of *Plasmodium falciparum*.

## Data Availability

The data used to support the findings of this study are available from the corresponding author upon request.

## Abbreviations

BMI: Body mass index
CER-ISBA: Research ethic committee of apply biomedical sciences institute
COPHARBIOTEC: Coopération Pharmaceutique Biologique et Technique
CREAT: Creatinine
CTCAE: Common terminology criteria for adverse events
DBP: Diastolic blood pressure
GOT: Glutamic oxaloacetic transaminase
GPT: Glutamic pyruvic transaminase
GLY: Glycaemia
Hb: Haemoglobin
J: Day
LQAS: Lot Quality Assurance of Sampling
NR: Number of red cells
NW: Number of white cells
SBP: Systolic blood pressure
TP: Prothrombin rate.
